# *nala*: text mining natural language mutation mentions

**DOI:** 10.1093/bioinformatics/btx083

**Published:** 2017-02-13

**Authors:** Juan Miguel Cejuela, Aleksandar Bojchevski, Carsten Uhlig, Rustem Bekmukhametov, Sanjeev Kumar Karn, Shpend Mahmuti, Ashish Baghudana, Ankit Dubey, Venkata P Satagopam, Burkhard Rost

**Affiliations:** 1 TUM, Department of Informatics, Bioinformatics & Computational Biology – i12, Garching, Munich, Germany; 2 TUM Graduate School, Center of Doctoral Studies in Informatics and its Applications (CeDoSIA), Garching, Germany; 3 Microsoft, WA, Bellevue, USA; 4 Ludwig Maximilian University, 80538 Munich & Siemens AG, Corporate Technology, Munich, Germany; 5 BITS-Pilani K. K. Birla Goa Campus, Goa, India; 6 Concur (Germany) GmbH, Frankfurt am Main, Germany; 7 Luxembourg Centre for Systems Biomedicine (LCSB), University of Luxembourg, Belvaux, Luxembourg; 8 Institute of Advanced Study (TUM-IAS) & Institute for Food and Plant Sciences WZW – Weihenstephan & New York Consortium on Membrane Protein Structure (NYCOMPS) & Department of Biochemistry and Molecular Biophysics, Columbia University, New York, NY, USA

## Abstract

**Motivation:**

The extraction of sequence variants from the literature remains an important task. Existing methods primarily target standard (ST) mutation mentions (e.g. ‘E6V’), leaving relevant mentions natural language (NL) largely untapped (e.g. ‘glutamic acid was substituted by valine at residue 6’).

**Results:**

We introduced three new corpora suggesting named-entity recognition (NER) to be more challenging than anticipated: 28–77% of all articles contained mentions only available in NL. Our new method *nala* captured NL and ST by combining conditional random fields with word embedding features learned unsupervised from the entire PubMed. In our hands, *nala* substantially outperformed the state-of-the-art. For instance, we compared all unique mentions in new discoveries correctly detected by any of three methods (SETH, tmVar, or *nala*). Neither SETH nor tmVar discovered anything missed by *nala*, while *nala* uniquely tagged 33% mentions. For NL mentions the corresponding value shot up to 100% *nala*-only.

**Availability and Implementation:**

Source code, API and corpora freely available at: http://tagtog.net/-corpora/IDP4+.

**Supplementary information:**

Supplementary data are available at *Bioinformatics* online.

## Introduction

Genetic variations drive biological evolution. Yet, most mutations might harm (Rost, 1996; Rost *et al.*, 2003; Sawyer *et al.*, 2007). Experimental studies elucidating the effects of sequence variation remain precious and expansive. Today, the important results from such studies are still published in papers. Repositories, such as OMIM, rely primarily on labor-intensive and time-consuming expert curation. Searching PubMed with relevant keywords (http://1.usa.gov/1rCrKwR) brought up >1M articles; most of those (>630K) for variation in human. An equivalent search of UniProtKB/Swiss-Prot (Boutet *et al.*, 2016; UniProt, 2015) revealed ∼13K indexed publications, and the professional version of the Human Gene Mutation Database (HGMD) (Stenson *et al.*, 2003) listed ∼179K mutations. These numbers sketch the immense information gap between literature and database annotations (Jimeno and Verspoor, 2014a,b, Database). Despite two decades of high-level efforts to increase the incentive for authors to link their findings to databases, this gap is likely to expand even more rapidly in the future. Instead of requiring administrative overhead, the text mining of free literature pursues a solution that could scale and substantially narrow the gap (Krallinger *et al.*, 2008).

*Mutation mentions* refers to the format used to report experimental results for sequence variants. Mining mutation mentions is referred to as *named-entity recognition* (NER). We focused on the task to recognize and parse text fragments such as the following two equivalent mutation mentions: ‘glutamic acid was substituted by valine at residue 6’ or ‘p.6E > V’. The two differ only in their syntax: the first is written in natural language (NL), the second follows a standardized format (ST).

Existing extraction methods primarily target simple and standardized mutation mentions. MutationFinder (MF) (Caporaso *et al.*, 2007a,b) uses a large set of regular expressions (*regexes*) to recognize single nucleotide or amino acid variants written in simple ST form (e.g. ‘E6V’) and slightly more complex semi-standard (SST) form (e.g. ‘Glu 6 to Val’ or ‘glutamic acid for valine 6’). *SETH* (Thomas *et al.*, 2016) recognizes other short sequence variations such as insertions and deletions (*indels*, e.g. ‘c.76_77insG’ and ‘c.76delA’, resp.) by implementing a formal grammar and regexes that cover recommended, deviations and deprecated cases of the HGVS nomenclature (den Dunnen *et al.*, 2016). The HGVS nomenclature aims to frame mutation mentions in a canonical *normalized* language (e.g. the complete form ‘p.Glu6Val’ is preferred over alternatives). *tmVar* (Wei *et al.*, 2013) has introduced probabilistic methods and recognizes ST mentions for a large variety of variant types: point variants (SNVs: Single Nuclear Variants, SAVs: Single Amino acid Variants), structural variations (insertions, deletions, frameshifts: e.g. ‘p.(Arg97fs)’, duplications: e.g. ‘c.76dupA’), and *rsids* (reference SNP ID numbers, e.g. ‘rs206437’, i.e. dbSNP accession numbers (Sherry *et al.*, 2001)). None of these three methods appear to extract genetic markers (e.g. ‘D17S250’) nor large-scale mutations, i.e. variations of regions longer than a few nucleotides or amino acids (e.g. ‘TP73Δex2/3’ or ‘abrogated loss of Chr19’). Existing methods are reviewed in detail elsewhere (Jimeno and Verspoor, 2014a,b, F1000Res.; Nagel *et al.*, 2009). Mapping the variant E6V to a particular sequence, e.g. that of hemoglobin S in human with the SWISS-PROT identifier *hbb_human* and relating it to sickle cell anemia (SKCA) and finally identifying that the variants is actually at position 7 in the sequence, i.e. should have been named E7V (p.Glu7Val), are all essential steps toward ‘parsing the meaning’ of the annotation. We ignored these mapping problems in this work. Instead, our work focused on presenting the first comprehensive study of the significance of natural language mutation mentions (e.g. ‘in-frame deletion of isoleucine 299’). Our new method completed the picture by recognizing different mutation types (for both genes and proteins) written in simple form or complex natural language.

## 2 Materials and methods

### 2.1 Classification of mutation mentions: ST, SST and NL

There is no single reliable classification of natural language (NL) or standard (ST) mutation mentions. Some annotators might consider *‘alanine 27 substitution for valine’* as NL because it does not follow the standard HGVS nomenclature. Others might consider it as standard or semi standard (SST) because simple regexes might capture this mention. Previous mutation extraction methods primarily used regexes and did not capture long mutation mentions.

As an operational definition, we considered any long mention that was not recognized by previous methods as NL, any mention that resembled the HGVS nomenclature as ST, and any mention in between as SST. We defined the following if-else chain algorithm to capture this idea: given a mutation mention, if it matches custom regexes or those from tmVar, then it is ST; else if it has 5 or more words or contains 2 or more English-dictionary words, then it is NL; else if it contains 1 English-dictionary word, then it is SST; else it is ST (examples in Table 1). Our custom regexes matched one-letter-coded mentions such as ‘p.82A > R’ or ‘IVS46: del T -39 … -46’ (Supplementary Table S9). The collected *tmVar* regexes were used by the authors (Wei *et al.*, 2013) as features of the *tmVar* probabilistic model and as post-processing (PstPrc) rules.

**Table 1:** Classification of mutation mentions

*Class*	*Examples*	*MF*	*SETH*	*tmVar*
*ST*	Q115P; Asp8Asn; 76A>T c.925delA; g.3912G>C; rs206437 c.388 + 3insT delPhe1388; F33fsins; IVS3(+1); D17S250; TP73Δex2/3	yes no no no	yes yes no no	yes yes yes no
*SST*	3992-9g–>a mutation; codon 92, TAC–>TAT Gly 18 to Lys; leucine for arginine 90 G643 to A; abrogated loss of Chr19	no yes no	no yes no	yes no no
*NL*	glycine to arginine substitution at codon 20 glycine was substituted by lysine at residue 18 deletion of 10 and 8 residues from the N- and C-terminals	yes no no	yes no no	no no no

*Note*: Examples of mutation mentions of increasing level of complexity as found in the literature (*ST: standard*; *SST: semi-standard*; *NL: natural language*). The columns *MF*, *SETH* and *tmVar* indicate if the methods MutationFinder, SETH and tmVar, respectively, recognize the examples listed.

### 2.2 Evaluation measures

We considered a named entity as successfully *extracted* if its *text offsets* (character positions in a text-string) were correctly identified (tp: *true positive*). We considered two modes for *tp*: *exact* matching (two entities match if their text offsets are *identical*) and *partial* matching (text offsets *overlap*). Any other prediction was considered as a *false positive* (fp) and any missed entity as a *false negative* (fn). Partial matching is more suitable to evaluate NL mentions lacking well-defined boundaries. For instance, in finding *‘[changed conserved] glutamine at 115 to proline’*, we did not distinguish solutions with and without the words in brackets, because we focused on the extraction of the mention not on that of additional annotations (here ‘*conserved*’). We computed performance for all cases and for the subclasses (ST, SST and NL). A test entity of subclass X was considered as correctly identified if any predicted entity matched. We then used the standard evaluation measures for named-entity recognition, namely, *precision* (P: tp/tp+fp), *recall* (R: tp/tp+fn) and *F-Measure* (F: 2*(P*R)/(P+R)). Within a corpus, we computed the StdErr by randomly selecting 15% of the test data without replacement in 1000 (n) bootstrap samples. With *<x> *as the overall performance for the entire test set and *x_i_* for subset *i*, we computed:

(1) $$\sqrt{\frac{1}{n-1}\sum_{i=1}^{n}{\left(x_{i}-〈x〉\right)}^{2}}\;StdErr=\;\frac{\sigma }{\sqrt{n}}$$

Across corpora, we did not merge documents. Rather, we computed the mean of P, R and F between the considered corpora, and computed the StdErr of the mean without subsampling.

### 2.3 Previous corpora

Some well-known corpora annotate mutation mentions and specific text offsets, including: *SETH* (Thomas *et al.*, 2016), *tmVar* (Wei *et al.*, 2013) and *Variome* (Verspoor *et al.*, 2013). All corpora contain different mutation types, including SNPs, frameshifts, or deletions (primarily in ST or SST forms). *SETH* and *tmVar* annotated abstracts, *Variome* full-text articles. The *Variome* corpus annotated many vague mentions (e.g. ‘*de novo* mutation’ or ‘large deletion’). With *Variome120* we referred to a *Variome* subset of position-specific variants with 118 mentions as described earlier (Jimeno and Verspoor, 2014a,b, F1000Res.) plus two new annotations with reference to both a DNA and a protein mutation.

### 2.4 Three new corpora: *IDP4*, *nala* and *nala_discoveries*

We annotated three new corpora (*IDP4*, *nala* and *nala_discoveries*) at different times and with slightly different objectives. These solutions substantially enriched the *status quo*. All three were annotated with the tool *tagtog* (Cejuela *et al.*, 2014). The differences were as follows.

#### 2.4.1 IDP4 corpus

We introduced the *IDP4* corpus to offer an unbiased representation of mutation mention forms (NL in particular). Previous corpora focused on ST or SST mentions. We annotated the entities *Mutation*, *Organism* and *GGP* (gene or gene product), as well as, relations between *GGP* and both *Mutation* and *Organism*. We included abstract-only and full-text documents. Documents were selected in four steps. (1) Include particular organisms/sources (*Homo sapiens, Arabidopsis thaliana, Drosophila melanogaster, Caenorhabditis elegans, Schizosaccharomyces pombe, Saccharomyces cerevisiae, Mus musculus, Rattus norvegicus* and *HIV*). (2) Collect the PubMed identifiers linked from SWISS-PROT (Boutet *et al.*, 2016) that cite the keywords variation or mutagenesis. (3) Accept all abstracts that contain any of five keywords (*mutation, variation, insertion, deletion, SNP*). (4 optionally) Retrieve full-text articles through keyword *open access* (on PubMed Central).

Our method and thus our annotation guidelines needed mutation mentions with three components: (1) W (word): a clear word or pattern giving the variant and its type (W is binary, i.e. present or not), e.g. W = yes as in ‘His72 *substitution to* Arg’ or ‘24bp *duplication* of ARX exon 2’. (2) L (letter): giving the mutated nucleotides or residues (L is binary, i.e. present or not), e.g. L = yes as in ‘delta Phe581’ and L = no as in ‘deletion at pos. 581’. (3) P (position): giving the sequence location of the variation (P has three values: exact, vague, or no, i.e. not applicable), e.g. P = exact as in ‘Tyr838 mutation’ or ‘Del 1473-IVS16(+2)’ and P = vague as in ‘placed immediately downstream of I444’ or ‘at the carboxyl end’.

We annotated two cases: (1) W = yes, L = yes, P = yes|vague, e.g. ‘p.Phe54Ser’, ‘Arg-Thr insertion between 160 and 161 residues’, or ‘(499)leucine (TTA) to isoleucine (ATA)’; (2) W = yes, L = no, P = yes, e.g. ‘point mutation at amino acid 444’, ‘SNPs affecting residues, 282, 319 and 333’. The rationale was that we could assign to the missing nucleotide/residue the unknown value *X*. We also annotated total gene knockouts (‘Δ/Δ’), deletions of subparts (‘deleted C1 domain’), or deletions of larger regions (‘deletions of chromosome 9p22.3’). We considered those positions as specific. Moreover, we annotated rsids.

We measured the agreement between annotators (F-Measure of the inter-annotator agreement: *F_IAA*) as proxy for the consistency of the annotations. Four annotators participated. Across 53 overlapping documents, for IDP4 we observed F_IAA = 91 for all mutation mentions and F_IAA = 77 for NL mentions. In total, the *IDP4* corpus collected 157 documents (72 full text + 85 abstracts) with 3337 mutation annotations: 3113 ST mentions (93%), 198 NL (6%) and 26 SST (1%).

#### 2.4.2 nala corpus

We introduced the *nala* corpus to expand the amount of NL mutation mentions necessary for the training of probabilistic methods. No previous corpus tagged enough (Results) (Ravikumar *et al.*, 2012). We annotated only abstracts for they contained higher densities (number of mentions/number of words) of NL mentions than full articles. In particular, the *IDP4*, *Variome* and *Variome120* corpora contained more NL mentions per word in abstracts than in full texts (ratios: 5.5, 1.6 and 3.8). We selected documents as for the *IDP4* corpus but applied *active learning* to simultaneously build corpus and method (details below). The *nala* corpus consisted of two disjoint sets: *nala_training* and *nala_known*. The latter ‘blind’ set with 90 randomly chosen abstracts (15% of the entire *nala* corpus) was used only to test. We stopped adding abstracts to this test set when the standard error estimate plateaued. Moreover, *nala_known* contained 8 documents (9% of test) without any annotation, i.e. no mutation mentions, to effectively probe the precision of methods.

Annotating NL mentions strictly following our *IDP4* corpus guidelines was more challenging. For example, mutation positions were often vague and/or referenced indirectly in other sentences than the variant and often in different paragraphs. In particular, we relaxed the rules more for insertions and deletions, e.g. ‘2-bp deletion in exon 6’, ‘somatic 16-bp deletion’, or ‘in-frame insertion of 45 nucleotides’. Another unique feature of the *nala* corpus was the annotation of genetic markers. To limit the workload, for the *nala* corpus we refrained from annotating organisms or GGP terms. Only to ease the reading of mutation mentions, we used the GNormPlus tagger (Wei *et al.*, 2015) to automatically annotate gene/protein terms.

Three experts annotated *nala*; their agreement over 30 documents was F_IAA = 95 for all mutation mentions and F_IAA = 89 for NL. The *nala* corpus collected 591 abstracts with 2108 mutation annotations. Despite the explicit focus on NL mentions, ST mentions still dominated (presumably because they are easier to annotate): 1097 ST (52%) versus 841 NL (40%) and 170 SST (8%). As a result, the *nala_known* set benchmarked both ST and NL mentions (SST mentions were underrepresented).

#### 2.4.3 nala_discoveries corpus

We introduced another novel corpus, *nala_discoveries*, to gauge automatic tagging of papers with ‘new discoveries’. The idea is best explained in comparison to our generic *nala* corpus: there we picked the PubMed articles beginning from identifiers of genes and proteins that had already been described experimentally and annotated in SWISS-PROT (Boutet *et al.*, 2016). We had not realized how crucial this constraint was until we created a new corpus just before submitting the manuscript. The usage of previously-indexed articles and knowledge has been common practice, e.g. for SNPs indexed by *dbSNP* or HGVS-compliant mentions (*SETH* corpus), disease- and mutation-specific MeSH terms indexed by PubMed (*tmVar* corpus), mutation-specific citations indexed by SWISS-PROT (*IDP4* and *nala*). Only the *Variome* corpus directly searched PubMed, but it was limited to three Lynch syndrome genes. For *nala_discoveries*, we found all articles in PubMed using the keyword *mutation* and published between 2013 and 2016 in the journals Nature, Science and Cell, without further filtering (exact search: http://bit.ly/2aHthKP). To limit the workload, we randomly selected abstracts with at least one mutation mention (any form) and stopped at 60 abstracts with *at least one NL* mention. We applied the guidelines used for *IDP4* and *nala*. Compared to other corpora, we found more large-scale mutations (e.g. chromosomal translocations) and significant differences in the semantics of mutation mentions. The numbers for *nala_discoveries* were: 78 abstracts (18 with ST or SST mentions only) and 215 mutation annotations spanning 104 ST mentions (48%), 71 NL (33%) and 40 SST (19%). The corpus *nala_discoveries* effectively benchmarked all mention classes (incl. SST) and was annotated by the same three annotators as the *nala* corpus.

### 2.5 New method: *nala*

The new method *nala* was based on conditional random fields (CRFs) (Lafferty *et al.*, 2001). Techniques for CRFs are amply described (Settles and Burr, 2004; Wei *et al.*, 2013; Wei *et al.*, 2015). We used the *python-crfsuite* implementation, a python binding of the *CRFSuite* C ++ library (software URLs in Supplementary Table S10). We used our in-house implementation of the *tmVar tokenizer* (Wei *et al.*, 2013), but did not split tokens upon case changes at the sentence beginning (‘The’ not ‘T’+ ’he’). We applied *BIEO* token labeling: tokens at the *beginning* of a mutation mention were labeled as *B*; continuing (*inside*) tokens as *I*; *ending* tokens as *E*; all other tokens (*outside* a mention) as *O*. For NL, *BIEO* outperformed our implementation of the 11 *tmVar* labels. We also included standard features such as token stems, word patterns, prefix and suffix characters, presence of numbers, or the word belonging to term dictionaries such as nucleotides, amino acids, or other common entities. We also added PstPrc rules such as fixing small boundary problems (‘+1858C > T’ not ‘1858C > T’). Finally, we introduced two optional post-processing (PstPrc) regex-based filters that can be switched on or off by users: 1) annotate rsids or not, and 2) annotate genetic markers or not.

Word embedding features (WE) contributed most to our new method. *WE* features had already helped in biomedical named-entity recognition (Guo *et al.*, 2014; Passos *et al.*, 2014; Seok *et al.*, 2016; Tang *et al.*, 2014). Specifically, we used neural networks with the CBOW architecture (continuous bag of words) (Mikolov *et al*., 2013) and trained on all PubMed abstracts until mid 2015. We used window = 10 and dimension D = 100. Tokens were converted to lowercase and digits were normalized to 0. For each token, the vector of 100 real values was translated into 100 features. The real values were used as weights in the CRF features, e.g.: word_embedding[0]=0.00492302. In analogy to the optional PstPrc filters, users also have the option to run *nala* with WE features (default) or not (the features are not computed).

**Fig. 1:**
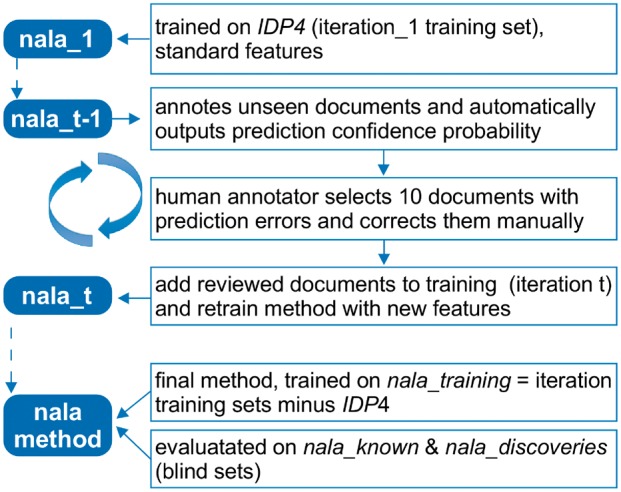
*nala* method active learning process. Each blue box represents an iteration state of the *nala* method. The method and the iteration training sets are implemented in parallel. The previous iteration method (*nala_t-1*) is used to automatically annotate unseen documents. Selected documents with outstanding errors are reviewed manually and added to the iteration training set *t*. New features are evaluated in 5-fold cross validation and the method is retrained with all previous sets (*nala_t*). At the end, the sum of iteration training sets without *IDP4* form the *nala_training* corpus. The final *nala* method is trained on *nala*_training (only) and evaluated against the *nala_known* and *nala*_discoveries corpora

We built the *nala* corpus and method in parallel through iterative active learning (Fig. 1). We implemented a base version (*nala_1)* using the features from *tmVar* and trained on the *IDP4* corpus (*iteration_1* training set). For later iterations (*iteration_t*), we used the previous model (*nala_t-1*) and a high-recall set of regexes to select documents with non-ST mentions. We selected only documents with ≥1 NL mention. In each iteration, we arbitrarily selected ten documents. These were pre-annotated by *nala_t-1* and then posted to the *tagtog* annotation tool for expert review and refinement; the reviewed annotations were saved as *iteration_t*. In each iteration step, we trained through 5-fold cross-validation. Annotators selected documents with annotation errors (missing entities, wrong offsets, or false positives) to learn those. In the end, the merging of iteration sets without *IDP4* created the *nala_training* corpus. We trained the final method solely on *nala_training* (without using *IDP4* as training data), due to two reasons. Firstly, NL mentions were learned much better with *nala_training*. Secondly, ST mentions were learned better including *IDP4*, yet the small improvement did not justify the complexity of two separate models (ST and NL). We used *nala_known* and *nala_discoveries* only to evaluate the final method.

### 2.6 Methods for comparison

We compared *nala* with two state-of-the-art methods, namely *SETH* and *tmVar*. To run *SETH* locally, we slightly modified the original *scala* code to print out the results in *brat format*. To run *tmVar*, we used its official API. We could not benchmark the *tmVar* API on the *tmVar* test set, as it had been trained on this set. For each method, we evaluated its default and its *best* performance. To compute the *best* performance, we filtered out some test annotations and predictions originating from arbitrary annotation guidelines of the individual corpora. For example, the *best* performance of *tmVar* on the *SETH* corpus disregarded rsids; *tmVar* predicts rsids but the *SETH* corpus does not consistently annotate them (9 out of 69). Analogously, *nala* predicted many NL mentions not annotated in the *SETH*, *tmVar*, or *Variome120* corpora. Overall, we applied the two PstPrc filters (rsids and genetic markers) and the usage or not of WE features (only for *nala*). WE features improved the performance for NL mentions (details below) but without WE features *nala* did better on the ST-scoped corpora. For all methods, the difference between default and *best* performance was consistently and substantially larger than the standard error within the corpus. This underlined the significance of annotation guidelines. Consequently, we reported (Results) the averages for default and best performance and their standard errors (individual results in Supplementary Tables S1–S5).

## 3 Results and discussion

### 3.1 Natural language (NL) mutation mentions important

**Fig. 2:**
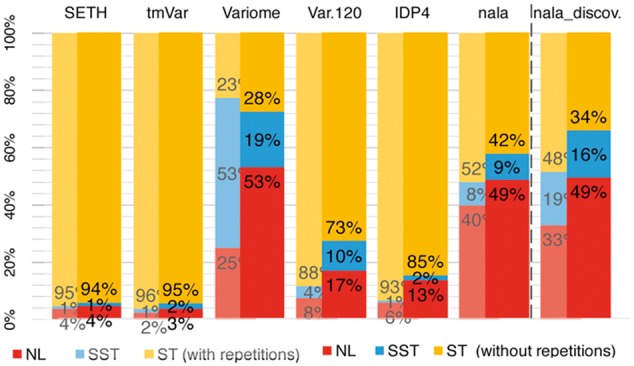
Natural language (NL) mutation mentions important. What type of mutation mentions dominates annotated corpora that somehow sample the literature: standard (ST, e.g. E6V), semi-standard (SST), or natural language (NL)? Grayed out bars indicate counts with repetitions, full bars unique mentions (e.g. E6V occurring twice in the same paper, is counted twice for the grayed out values and only once per paper for the others). The *Variome*, *Variome120*, *IDP4* and *nala_discoveries* corpora assembled different representations of NL mentions. The dashed line separates corpora with papers describing well-known, well-indexed genes and proteins (left of dashed line: *SETH*, *tmVar*, *Variome*, *Variome120*, *IDP4* and *nala_known*) and articles describing more recent discoveries that still have to be indexed in databases (right of dashed line: *nala_discoveries*) (Color version of this figure is available at *Bioinformatics* online.)

The *Variome120* and *IDP4* corpora (no bias in mention forms) had much higher fractions of NL over ST or SST mentions (8% and 6%, respectively; Fig. 2, grayed out bars) than *SETH* (4%) and *tmVar* (2%). Removing repetitions, the fraction of unique NL mentions increased to 17% and 13% (Fig. 2, highlighted bars). The *Variome* corpus contained the largest fraction of SST mentions (53% with and 19% without repetitions). NL mentions dominated abstracts even more (12% in *Variome120* and 13% in *IDP4* with mention repetitions and 29% and 17% without repetitions). The *nala* corpus, introduced here, was built with a higher fraction of NL mentions (40% with repetitions and 49% without repetitions). All these corpora relied on well-annotated genes and proteins (indexed articles). In contrast, the *nala_discoveries* corpus randomly sampled abstracts without considering previous functional annotations (no previous indices). It contained the largest percentage of combined NL + SST mentions (52% with repetitions and 65% without repetitions).

How many experimental results will methods miss from the three corpora (*IDP4*, *Variome* and *Variome120*) that focus on ST or SST mentions? 28–36% of all abstracts contained at least one NL mention not in ST form (Table 2). The corresponding per-mention fractions were 13–27% (Table 2). For *nala_discoveries* the numbers were substantially higher: 67–77% (per-document) and 43–51% (per-mention).

**Table 2:** Significance of NL mentions

	IDP4		Variome	Var.120	nala_discoveries		
Annotator*	(1)	(2)			(1)	(2)	(3)
Documents	30%	42%	22%	33%	78%	62%	77%
Mentions	14%	19%	6%	40%	52%	39%	49%

*Note*: Percentages of documents (3^rd^ row) or mentions (4^th^ row) that contain at least one NL (natural language) or SST (semi-standard) for which no ST (standard) mention exists in the same text. *Two different annotators were compared for the corpus *IDP4*; three different annotators were compared for the corpus *nala_discoveries*.

### 3.2 New method *nala* performed top throughout

**Fig. 3:**
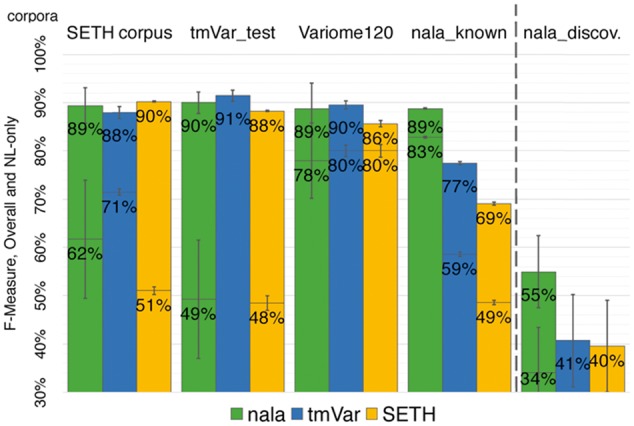
*nala* performed well for all corpora. The bars give two different results: values above the horizontal lines in bars reflect the F-measures for all mentions, while values below the horizontal lines in bars reflect the F-measures for the subset of NL-mentions in the corpus (high error bars indicate corpora with few NL mentions). The exception was the result for the method tmVar on the corpus tmVar_test, which was taken from the original publication of the method in which no result was reported for NL-only (Wei *et al.*, 2013). That publication reports only *exact matching* performance, i.e. its *overlapping* performance might be higher than shown here. *nala* consistently matched or outperformed other top-of-the-line methods in well-indexed corpora (*SetsKnown;* left of dashed line) and substantially improved over the *status quo* in recent non-indexed discoveries (*nala_discoveries*; right of dashed line). The F-measures of tmVar and SETH for NL-only on *nala_discoveries* was essentially zero (two rightmost bars) (Color version of this figure is available at *Bioinformatics* online.)

In our hands, the new method *nala* compared favorably with existing tools for extracting standard (ST) mutation mentions and significantly outperformed the status-quo for natural language (NL) mutation mentions (Fig. 3). This baseline was valid for all evaluations that we carried out. We found it more difficult to yield a single answer for the performance of *nala* (and from *nala* compared to other methods) because the performance depended crucially on the corpus. Each corpus has its own focus and bias. Which one best reflects what users expect?

We tried to simplify by grouping results into those for previously indexed mutations (*SetsKnown* corpora: *SETH*, *tmVar_test*, *Variome120* and *nala_known*; Supplementary Table S6) and those without prior knowledge (*nala_discoveries*; Supplementary Table S5). To establish the performance on well-annotated genes and proteins, the *SetsKnown* corpora might provide the least biased estimate: the *nala* method overall obtained F = 89 ± 3 compared to the highest performing competitor, i.e. *tmVar* with F = 87 ± 3 (Table 3). In contrast, the *nala_discoveries* corpus best established how well text mining works for new articles: the *nala* method reached F = 55 ± 7 compared to the highest performing competitors *SETH* and *tmVar* with F = 41 ± 10 (Table 3). Precision was very high for all methods on all evaluations and always lower than recall (for *nala* avg. on *SetsKnown* P = 87/R = 92; on *nala_discoveries* P = 90/R = 40). Thus, precision is a proxy for the performance on documents without mutation.

**Table 3:** Previously indexed versus new discoveries

	*SetsKnown* (indexed texts)			*nala_discoveries* (no indices)		
method	P	R	F ± StdErr	P	R	F *±* StdErr
*nala*	87	92	89 ± 3	90	40	55 ± 7
*tmVar*	95	79	87 ± 3	93	26	41 ± 10
*SETH*	97	74	83 ± 5	93	25	40 ± 10

*Note*: Precision (P), Recall (R) and F-Measure (F) for methods on corpora with previously indexed articles (*SetsKnown*: *SETH*, *tmVar_test*, *Variome120*, *nala_known*) and a corpus directly sampled from PubMed without index (*nala_discoveries*).

**Fig. 4:**
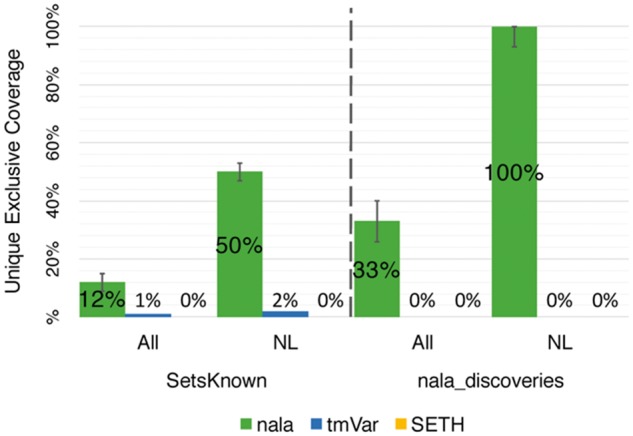
*nala* could fully replace other methods. For each publication we considered all mentions correctly identified by one of the top three methods and kept only the findings unique in each publication. The y-axis plots the percentage of those mentions identified uniquely by one of the methods (All: all mentions, NL: NL-only mentions). For all corpora containing publications of genes and proteins indexed in the databases (*SetsKnown*), 1% of the mentions were detected only by *tmVar* and 12% only by *nala*, while *SETH* found no mention in this dataset that *nala* had not detected. Only *nala* correctly detected NL-only mentions in abstracts with new discoveries (100% bar on right triplet)

Our new method *nala* essentially constituted a superset for the other two top methods in the following sense. The mutations correctly detected by *tmVar* and *SETH* were also found by *nala*. On top, *nala* correctly detected many mutations that had been missed by both other methods (Supplementary Fig. S1). Specifically, we looked at the subset of mentions correctly detected by any of the three methods (without considering repetitions, i.e. counting the detection of E6V only once per publication): 12% (*SetsKnown* corpora) and 33% (*nala_discoveries*) of mentions were exclusively found by *nala* (Fig. 4). In contrast, only 1% and 0% (*SetsKnown* and *nala_discoveries)* were exclusively found by *tmVar*; *SETH* added no exclusive detection. Moreover, 50% (*SetsKnown*) and 100% (*nala_discoveries*) of NL mentions were exclusively found by *nala* and only *tmVar* found 2% of novel NL mentions in the *SetsKnown*.

### 3.3 WE features are crucial/large variants are challenging

**Fig. 5:**
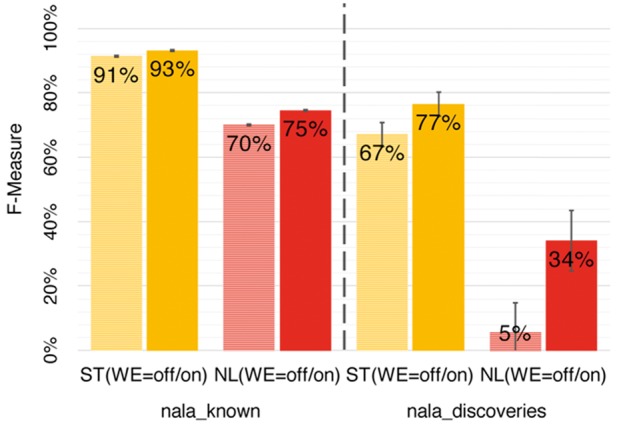
Word embedding (WE) features crucial for success. The inclusion of WE features (WE = on versus WE = off) substantially improved performance for both *nala_known* (texts previously indexed) and *nala_discoveries* (no previous indices)*.* The increase in performance was highest for NL mentions, but for ST mentions it was also significant

The Word Embedding (WE) features contributed significantly to the success of *nala* (Fig. 5). WE features improved performance for all mention types, most importantly for NL mentions (from F(WE = off)=70 to F(WE = on)=83 on *nala_known* corpus *and from* F(WE = off)=5 to F(WE = on)=34 on *nala_discoveries corpus*). In particular, WE vastly improved recall and even slightly improved the precision (Supplementary Table S8). All other features by the *nala* method were specific to mutation mentions and resulted from a laborious expert optimization. In contrast, WE features leveraged unsupervised data, i.e. can be adopted with minor modifications to any task or corpus.

We studied NER – Named Entity Recognition and ignored the considerably more difficult problem to map mutation mentions to sequences as needed to curate databases. Recent methods aim at this end (Mahmood *et al.*, 2016; Ravikumar *et al.*, 2015; Vohra and Biggin, 2013). However, all methods still primarily target SNVs/SAVs. We plan to extend the new corpora with exhaustive mapping annotations and to adapt the *nala* method to better cope with large-scale variations (predominant in *nala_discoveries*).

On new discoveries, the recall was 40%, i.e. 60% of the annotations were missed. 70% of these were large-scale variants, i.e. variations of regions longer than a few nucleotides or amino acids (presumably because their descriptions were less well-defined). For 44 of the 70% missed annotations, the annotators succeeded to position the sequence region (e.g. ‘Deletion of the class 2 KNOTTED1-LIKE HOMEOBOX’ or ‘Robertsonian translocation between chromosomes 15 and 21’ or ‘amplification of 3q26/28 and 11q13/22’). For the remaining 26 of the 70% the descriptions of the variants were so vague that we could not assign sequences, but recognized large chromosomal changes (e.g. ‘DNA double-strand breaks’ or ‘copy-number variants’). To complete the analysis of the 60% annotations missed in *nala_discoveries*: 22 of the ‘small variation’ 30% (100-70 = 30) were SAVs and SNVs, and 8% were other short variants such as insertions, deletions and frameshifts involving only a few nucleotides. This implied that methods missed at least 2-3 times more single variants (SAVs and SNVs) in *nala_discoveries* than in *SetsKnown*, i.e. in proteins without previous annotations (data not shown; cf. 92% recall on *SetsKnown*, i.e. 8% missed annotations). As a practical use, we plan to research the performance of *nala* to effectively map HIV mutation mentions from whole PubMed (Davey *et al.*, 2014).

## 4 Conclusion

Previous accounts (Jimeno and Verspoor, 2014a,b, F1000Res.; Thomas *et al.*, 2016; Wei *et al.*, 2013) suggested that the strict named-entity recognition (NER) of mutation mentions constitutes a solved problem with performance levels reported to be F > 85. Despite this optimism, the same authors (Caporaso *et al.*, 2007a,b; Jimeno and Verspoor, 2014a,b, Database) observed that methods failed to identify many mutations for database curation. Our work shed some light on this apparent paradox. First, mutation mentions often use natural language (NL) and were often missed by existing tools as they focused on standard (ST) forms. Second, existing corpora and methods primarily treated articles that had been previously indexed in databases. We showed that the percentage of publications with at least one mention in only NL ranged from 28 to 36% for indexed articles (*SetsKnown*) while it was twice as high (67–77%) for new discoveries (*nala_discoveries*, Table 2). Thus, most mentions relevant for database curation are only captured by methods versatile in NL.

We introduced the method *nala* designed to handle NL and ST mentions. In particular, word embedding (WE) features boosted performance for NL mentions (Fig. 5). In our hands, *nala* at least matched the best existing tools for publications that have already been curated in databases (corpora *SetsKnown*, dominated by ST mentions (F(nala)=89 ± 3 vs. F(tmVar)=87 ± 3, Table 3). Randomly sampling PubMed for new discoveries (*nala_discoveries*), *nala* was substantially better than existing methods (F(nala)=55 ± 7 versus F(SETH, tmVar)=40-41 ± 10, Table 3).

What do users have to expect: F = 89 or F = 55? The answer depends on what is known about the genes/proteins you are looking for. For *older* articles, point mutations, or *indels*, the current performance of all methods may suffice. For novel work or large-scale mutations, *nala* identifies many mutation mentions that are missed by others (Fig. 4). However, *nala* still missed about half of all variants described in the literature.

An important contribution of this work was the addition of three new corpora (*IDP4*, *nala_known* and *nala_discoveries*). These three new corpora accumulated the largest collection of mutation mentions: 826 documents (72 full texts), 627,953 tokens and 5660 mutation annotations (1110 NL). In comparison, the previous *SETH*, *tmVar* and *Variome120* corpora combined collect: 1,140 documents (10 full texts), 355,518 tokens and 2,933 mutation annotations (216 NL). In other words, this work boosted the available resources manifold. We released the new method as an open source python library and as API service and made the new corpora freely available: http://tagtog.net/-corpora/IDP4+

## Supplementary Material

Supplementary Data
