# Supplementary material for: nala: text mining natural language mutation mentions
Source: Bioinformatics. 2017 Feb 13;33(12):1852–8. doi: 10.1093/bioinformatics/btx083 (PMC5870606; doi:10.1093/bioinformatics/btx083)
Supplement: Supplementary Data [file btx083_supp.pdf]

Supporting online material
for:

nala: text mining of natural language mutations mentions

Juan Miguel Cejuela, Aleksandar Bojchevski, Carsten Uhlig, Rustem Bekmukhametov, Sanjeev Kumar Karn, Shpend Mahmuti, Ashish Baghudana, Ankit Dubey, Venkata P. Satagopam, & Burkhard Rost

1 Short description of Supporting Online Material

Some results not shown in main paper but supporting some described findings.

2 Material

(starts in next page; one Table/Figure per page)

Table S1. Individual results of all methods (default and *best* performances) on the *SETH* corpus (Thomas, et al., 2014). In bold the values considered for the final manuscript: averages of the default and *best* partial F-Measures and possibly maximum standard errors.

SETH corpus	Exact				Partial					
ST	P	R	F	StdErr	P	R	F	StdErr	Avg. F	Avg. StdErr
SETH	0.9195	0.8875	0.9032	0.0012	0.9333	0.8981	0.9154	0.0011		
SETH_best	0.9195	0.8875	0.9032	0.0012	0.9333	0.8981	0.9154	**0.0011**	**0.9154**	0.0000
tmVar	0.8994	0.8190	0.8573	0.0017	0.9158	0.8341	0.8731	0.0017		
tmVar_best	0.9601	0.8171	0.8828	0.0016	0.9769	0.8324	0.8989	0.0015	**0.8860**	**0.0129**
nala	0.7769	0.9049	0.8360	0.0016	0.8398	0.9727	0.9014	0.0012		
nala_best	0.9379	0.8759	0.9058	0.0010	0.9786	0.9206	0.9487	0.0007	**0.9251**	**0.0237**
NL	P	R	F	StdErr	P	R	F	StdErr	Avg. F	Avg. StdErr
SETH	0.9091	0.2941	0.4444	0.0089	1.0000	0.3429	0.5106	0.0080		
SETH_best	0.9091	0.2941	0.4444	0.0089	1.0000	0.3429	0.5106	**0.0080**	**0.5106**	0.0000
tmVar	0.8235	0.4242	0.5600	0.0085	1.0000	0.5556	0.7143	0.0066		
tmVar_best	0.8235	0.4242	0.5600	0.0085	1.0000	0.5556	0.7143	**0.0066**	**0.7143**	0.0000
nala	0.1071	0.4545	0.1734	0.0040	0.3291	1.0000	0.4952	0.0052		
nala_best	0.1897	0.3333	0.2418	0.0078	0.6184	0.9216	0.7402	0.0059	**0.6177**	**0.1225**
All	P	R	F	StdErr	P	R	F	StdErr	Avg. F	Avg. StdErr
SETH	0.9196	0.8597	0.8886	0.0012	0.9345	0.8713	0.9018	0.0011		
SETH_best	0.9196	0.8597	0.8886	0.0012	0.9345	0.8713	0.9018	0.0011	0.9018	0.0000
tmVar	0.8959	0.7998	0.8451	0.0018	0.9186	0.8208	0.8670	0.0018		
tmVar_best	0.9545	0.7978	0.8691	0.0017	0.9777	0.8191	0.8914	0.0015	0.8792	0.0122
nala	0.6818	0.8816	0.7689	0.0017	0.7656	0.9736	0.8571	0.0013		
nala_best	0.8779	0.8507	0.8640	0.0012	0.9421	0.9185	0.9302	0.0009	0.8937	0.0366

Table S2. Individual results of all methods (default and *best* performances) on the *tmVar_test* corpus (Wei, et al., 2013). In bold the values considered for the final manuscript: averages of the default and *best* partial F-Measures and possibly maximum standard errors. * tmVar could not be tested on tmVar_test and its results were taken from those reported in (Wei, et al., 2013). All following calculations use the reported exact performance results of tmVar instead of ignoring them, which in turn increased the overall performance of tmVar on *SetsKnown*.

tmVar_test	Exact				Partial					
ST	P	R	F	StdErr	P	R	F	StdErr	Avg. F	Avg. StdErr
SETH	0.9671	0.7933	0.8716	0.0019	0.9947	0.8198	0.8988	0.0016		
SETH_best	0.9671	0.7933	0.8716	0.0019	0.9947	0.8198	0.8988	**0.0016**	**0.8988**	0.0000
tmVar										
tmVar_best										
nala	0.8131	0.8112	0.8121	0.0022	0.9471	0.9641	0.9555	0.0010		
nala_best	0.9014	0.8427	0.8711	0.0019	0.9823	0.9310	0.9560	0.0009	**0.9558**	**0.0002**
NL	P	R	F	StdErr	P	R	F	StdErr	Avg. F	Avg. StdErr
SETH	0.3333	0.2353	0.2759	0.0198	0.6154	0.4000	0.4848	0.0150		
SETH_best	0.3333	0.2353	0.2759	0.0198	0.6154	0.4000	0.4848	**0.0150**	**0.4848**	0.0000
tmVar										
tmVar_best										
nala	0.0700	0.4118	0.1197	0.0044	0.2273	1.0000	0.3704	0.0051		
nala_best	0.1190	0.2941	0.1695	0.0086	0.4528	0.9600	0.6154	0.0058	**0.4929**	**0.1225**
All	P	R	F	StdErr	P	R	F	StdErr	Avg. F	Avg. StdErr
SETH	0.9471	0.7716	0.8504	0.0019	0.9820	0.8008	0.8822	0.0016		
SETH_best	0.9471	0.7716	0.8504	0.0019	0.9820	0.8008	0.8822	**0.0016**	**0.8822**	0.0000
tmVar*	0.9138	0.9140	0.9139							
tmVar_best*	0.9138	0.9140	0.9139						**0.9139**	**0.0000**
nala	0.6571	0.7931	0.7188	0.0023	0.8025	0.9660	0.8767	0.0015		
nala_best	0.8102	0.8190	0.8146	0.0021	0.9114	0.9329	0.9220	0.0011	**0.8994**	**0.0227**

Table S3. Individual results of all methods (default and *best* performances) on the *Variome120* corpus (Jimeno Yepes and Verspoor, 2014). In bold the values considered for the final manuscript: averages of the default and *best* partial F-Measures and possibly maximum standard errors.

Var.120	Exact				Partial					
ST	P	R	F	StdErr	P	R	F	StdErr	Avg. F	Avg. StdErr
SETH	0.9398	0.7647	0.8432	0.0106	0.9770	0.8095	0.8854	0.0066		
SETH_best	0.9398	0.7647	0.8432	0.0106	0.9770	0.8095	0.8854	**0.0066**	**0.8854**	0.0000
tmVar	0.8627	0.8627	0.8627	0.0057	0.9266	0.9352	0.9309	0.0068		
tmVar_best	0.8627	0.8627	0.8627	0.0057	0.9266	0.9352	0.9309	**0.0068**	**0.9309**	0.0000
nala	0.6870	0.8738	0.7692	0.0119	0.7746	0.9821	0.8661	0.0098		
nala_best	0.8667	0.8922	0.8792	0.0093	0.9386	0.9817	0.9596	0.0053	**0.9129**	**0.0468**
NL	P	R	F	StdErr	P	R	F	StdErr	Avg. F	Avg. StdErr
SETH	0.6667	0.3077	0.4211	0.0100	1.0000	0.6667	0.8000	0.0133		
SETH_best	0.6667	0.3077	0.4211	0.0100	1.0000	0.6667	0.8000	**0.0133**	**0.8000**	0.0000
tmVar	0.6667	0.3077	0.4211	0.0095	1.0000	0.6667	0.8000	0.0121		
tmVar_best	0.6667	0.3077	0.4211	0.0095	1.0000	0.6667	0.8000	**0.0121**	**0.8000**	0.0000
nala	0.1071	0.2500	0.1500	0.0181	0.5405	1.0000	0.7018	0.0092		
nala_best	0.2667	0.3077	0.2857	0.0167	0.7826	0.9474	0.8571	0.0052	**0.7795**	**0.0777**
NL	P	R	F	StdErr	P	R	F	StdErr	Avg. F	Avg. StdErr
SETH	0.9213	0.6833	0.7847	0.0107	0.9794	0.7600	0.8559	0.0068		
SETH_best	0.9213	0.6833	0.7847	0.0107	0.9794	0.7600	0.8559	**0.0068**	**0.8559**	0.0000
tmVar	0.8440	0.7667	0.8035	0.0071	0.9250	0.8672	0.8952	0.0084		
tmVar_best	0.8440	0.7667	0.8035	0.0071	0.9250	0.8672	0.8952	**0.0084**	**0.8952**	0.0000
nala	0.5629	0.7833	0.6551	0.0105	0.7225	0.9857	0.8338	0.0062		
nala_best	0.7638	0.8083	0.7854	0.0096	0.9048	0.9779	0.9399	0.0039	**0.8869**	**0.0531**

Table S4. Individual results of all methods (default and *best* performances) on the *nala_known* corpus. In bold the values considered for the final manuscript: averages of the default and *best* partial F-Measures and possibly maximum standard errors.

nala_known	Exact				Partial					
ST	P	R	F	StdErr	P	R	F	StdErr	Avg. F	Avg. StdErr
SETH	0.9764	0.7209	0.8294	0.0040	0.9922	0.7356	0.8449	0.0040		
SETH_best	0.9764	0.7294	0.8350	0.0041	0.9922	0.7442	0.8505	0.0039	**0.8477**	**0.0028**
tmVar	0.9424	0.7616	0.8424	0.0043	0.9931	0.8045	0.8889	0.0037		
tmVar_best	0.9424	0.7706	0.8479	0.0040	0.9931	0.8136	0.8944	0.0035	**0.8917**	**0.0027**
nala	0.8389	0.7267	0.7788	0.0044	0.9884	0.8763	0.9290	0.0024		
nala_best	0.8389	0.7267	0.7788	0.0044	0.9884	0.8763	0.9290	0.0024	**0.9290**	**0.0000**
NL	P	R	F	StdErr	P	R	F	StdErr	Avg. F	Avg. StdErr
SETH	0.4242	0.0979	0.1591	0.0029	1.0000	0.3210	0.4860	0.0041		
SETH_best	0.4242	0.0979	0.1591	0.0029	1.0000	0.3210	0.4860	**0.0041**	**0.4860**	0.0000
tmVar	0.3571	0.1056	0.1630	0.0035	1.0000	0.4142	0.5858	0.0045		
tmVar_best	0.3571	0.1056	0.1630	0.0035	1.0000	0.4142	0.5858	**0.0045**	**0.5858**	0.0000
nala	0.4896	0.3310	0.3950	0.0036	0.9310	0.7459	0.8282	0.0023		
nala_best	0.4896	0.3310	0.3950	0.0036	0.9310	0.7459	0.8282	0.0023	**0.8282**	**0.0000**
NL	P	R	F	StdErr	P	R	F	StdErr	Avg. F	Avg. StdErr
SETH	0.8439	0.4294	0.5692	0.0038	0.9847	0.5302	0.6893	0.0031		
SETH_best	0.8439	0.4320	0.5714	0.0038	0.9847	0.5331	0.6918	0.0032	**0.6906**	**0.0012**
tmVar	0.7650	0.4513	0.5677	0.0039	0.9959	0.6312	0.7727	0.0028		
tmVar_best	0.7650	0.4540	0.5698	0.0040	0.9959	0.6345	0.7751	0.0030	**0.7739**	**0.0012**
nala	0.6755	0.5280	0.5927	0.0034	0.9658	0.8208	0.8874	0.0016		
nala_best	0.6755	0.5280	0.5927	0.0034	0.9658	0.8208	0.8874	**0.0016**	**0.8874**	0.0000

Table S5. Individual results of all methods (default and *best* performances) on the *nala_discoveries* corpus. In bold the values considered for the final manuscript: averages of the default and *best* partial F-Measures. * The reported standard errors for nala_discoveries consider those of *SetsKnown* + *nala_discoveries*, Supplementary Table S7.

nala_discov.	Exact				Partial					
ST	P	R	F	StdErr	P	R	F	StdErr*	Avg. F	Avg. StdErr*
SETH	0.8545	0.4519	0.5912	0.0078	0.9322	0.5093	0.6587	0.0082		
SETH_best	0.8545	0.4519	0.5912	0.0078	0.9322	0.5093	0.6587	0.0082	**0.6587**	0.0000
tmVar	0.8571	0.4615	0.6000	0.0082	0.9333	0.5185	0.6667	0.0079		
tmVar_best	0.8571	0.4615	0.6000	0.0082	0.9333	0.5185	0.6667	0.0079	**0.6667**	0.0000
nala	0.7385	0.4615	0.5680	0.0078	0.9494	0.6410	0.7653	0.0069		
nala_best	0.7385	0.4615	0.5680	0.0078	0.9494	0.6410	0.7653	0.0069	**0.7653**	0.0000
NL	P	R	F	StdErr	P	R	F	StdErr*	Avg. F	Avg. StdErr*
SETH	0.0000	0.0000	0.0000	0.0000	0.0000	0.0000	0.0000	0.0000		
SETH_best	0.0000	0.0000	0.0000	0.0000	0.0000	0.0000	0.0000	0.0000	**0.0000**	0.0000
tmVar	0.0000	0.0000	0.0000	0.0000	0.0000	0.0000	0.0000	0.0000		
tmVar_best	0.0000	0.0000	0.0000	0.0000	0.0000	0.0000	0.0000	0.0000	**0.0000**	0.0000
nala	0.4167	0.0704	0.1205	0.0042	0.8889	0.2105	0.3404	0.0066		
nala_best	0.4167	0.0704	0.1205	0.0042	0.8889	0.2105	0.3404	0.0066	**0.3404**	0.0000
All	P	R	F	StdErr	P	R	F	StdErr*	Avg. F	Avg. StdErr*
SETH	0.8545	0.2186	0.3481	0.0057	0.9322	0.2511	0.3957	0.0060		
SETH_best	0.8545	0.2186	0.3481	0.0057	0.9322	0.2511	0.3957	0.0060	**0.3957**	0.0000
tmVar	0.8596	0.2279	0.3603	0.0057	0.9344	0.2603	0.4071	0.0061		
tmVar_best	0.8596	0.2279	0.3603	0.0057	0.9344	0.2603	0.4071	0.0061	**0.4071**	0.0000
nala	0.6585	0.2512	0.3636	0.0052	0.9020	0.3948	0.5493	0.0058		
nala_best	0.6585	0.2512	0.3636	0.0052	0.9020	0.3948	0.5493	0.0058	**0.5493**	0.0000

Table S6. Individual results of all methods (default and *best* performances) on the *SetsKnown* corpus (merge of: SETH corpus, tmVar_test, Variome120, and nala_known). In bold the values considered for the final manuscript: averages of the default and *best* partial F-Measures and possibly maximum standard errors. * tmVar could not be tested on tmVar_test and its results were taken from those reported in (Wei, et al., 2013). All following calculations used the reported exact performance results of tmVar instead of ignoring them, which in turn increased the overall performance of tmVar on *SetsKnown*.

SetsKnown	Exact				Partial					
ST	P	R	F	StdErr	P	R	F	StdErr	Avg. F	Avg. StdErr
SETH	0.9507	0.7916	0.8619	0.0163	0.9743	0.8158	0.8861	0.0150		
SETH_best	0.9507	0.7937	0.8633	0.0155	0.9743	0.8179	0.8875	0.0138	**0.8868**	**0.0007**
tmVar	0.9015	0.8144	0.8541	0.0061	0.9452	0.8579	0.8976	0.0172		
tmVar_best	0.9217	0.8168	0.8645	0.0101	0.9655	0.8604	0.9081	0.0115	**0.9029**	**0.0052**
nala	0.7790	0.8292	0.7990	0.0154	0.8875	0.9488	0.9130	0.0191		
nala_best	0.8862	0.8344	0.8587	0.0277	0.9720	0.9274	0.9483	0.0068	**0.9307**	**0.0177**
NL	P	R	F	StdErr	P	R	F	StdErr	Avg. F	Avg. StdErr
SETH	0.5833	0.2338	0.3251	0.0667	0.9039	0.4327	0.5704	0.0768		
SETH_best	0.5833	0.2338	0.3251	0.0667	0.9039	0.4327	0.5704	0.0768	**0.5704**	**0.0000**
tmVar	0.6158	0.2792	0.3814	0.1163	1.0000	0.5455	0.7000	0.0622		
tmVar_best	0.6158	0.2792	0.3814	0.1163	1.0000	0.5455	0.7000	0.0622	**0.7000**	**0.0000**
nala	0.1935	0.3618	0.2095	0.0628	0.5070	0.9365	0.5989	0.1025		
nala_best	0.2663	0.3165	0.2730	0.0472	0.6962	0.8937	0.7602	0.0543	**0.6796**	**0.0807**
All	P	R	F	StdErr	P	R	F	StdErr	Avg. F	Avg. StdErr
SETH	0.9080	0.6860	0.7732	0.0713	0.9702	0.7406	0.8323	0.0486		
SETH_best	0.9080	0.6867	0.7738	0.0708	0.9702	0.7413	0.8329	**0.0480**	**0.8326**	0.0003
tmVar*	0.8547	0.7330	0.7826	0.0751	0.9465	0.7731	0.8622	0.0314		
tmVar_best*	0.8693	0.7331	0.7891	0.0765	0.9531	0.8087	0.8689	**0.0317**	**0.8656**	0.0034
nala	0.6443	0.7465	0.6839	0.0383	0.8141	0.9365	0.8638	0.0118		
nala_best	0.7819	0.7515	0.7642	0.0594	0.9310	0.9125	0.9199	0.0114	**0.8918**	**0.0281**

Table S7. Individual results of all methods (default and *best* performances) on *SetsKnown* corpus + *nala_discoveries* (merge of: SETH corpus, tmVar_test, Variome120, nala_known, and nala_discoveries). In bold the values considered for the final manuscript: possibly maximum standard errors. * tmVar could not be tested on tmVar_test and its results were taken from those reported in (Wei, et al., 2013).

SetsKnown+nala_discov.	Exact				Partial					
ST	P	R	F	StdErr	P	R	F	StdErr	Avg. F	Avg. StdErr
SETH	0.9315	0.7237	0.8077	0.0556	0.9659	0.7545	0.8406	0.0470		
SETH_best	0.9315	0.7254	0.8088	0.0557	0.9659	0.7562	0.8418	**0.0470**	0.8412	0.0006
tmVar	0.8904	0.7262	0.7906	0.2369	0.9422	0.7731	0.8399	0.2495		
tmVar_best	0.9056	0.7280	0.7984	0.2399	0.9575	0.7749	0.8477	**0.2522**	0.8438	0.0039
nala	0.7709	0.7556	0.7528	0.0477	0.8999	0.8872	0.8835	0.0331		
nala_best	0.8567	0.7598	0.8006	0.0620	0.9675	0.8701	0.9117	**0.0370**	0.8976	0.0141
NL	P	R	F	StdErr	P	R	F	StdErr	Avg. F	Avg. StdErr
SETH	0.4667	0.1870	0.2601	0.0831	0.7231	0.3461	0.4563	0.1286		
SETH_best	0.4667	0.1870	0.2601	0.0831	0.7231	0.3461	0.4563	**0.1286**	0.4563	0.0000
tmVar	0.4618	0.2094	0.2860	0.1506	0.7500	0.4091	0.5250	0.2357		
tmVar_best	0.4618	0.2094	0.2860	0.1506	0.7500	0.4091	0.5250	**0.2357**	0.5250	0.0000
nala	0.2381	0.3035	0.1917	0.0518	0.5834	0.7913	0.5472	0.0948		
nala_best	0.2963	0.2673	0.2425	0.0476	0.7347	0.7571	0.6763	**0.0939**	0.6117	0.0645
All	P	R	F	StdErr	P	R	F	StdErr	Avg. F	Avg. StdErr
SETH	0.8973	0.5925	0.6882	0.1014	0.9626	0.6427	0.7450	0.0951		
SETH_best	0.8973	0.5930	0.6886	0.1013	0.9626	0.6433	0.7455	**0.0950**	0.7452	0.0002
tmVar*	0.8557	0.6319	0.6981	0.1026	0.9435	0.6449	0.7712	0.0942		
tmVar_best*	0.8674	0.6321	0.7033	0.1042	0.9583	0.6453	0.7765	**0.0956**	0.7739	0.0027
nala	0.6472	0.6474	0.6198	0.0706	0.8317	0.8282	0.8009	0.0635		
nala_best	0.7572	0.6514	0.6841	0.0924	0.9252	0.8090	0.8458	**0.0746**	0.8233	0.0225

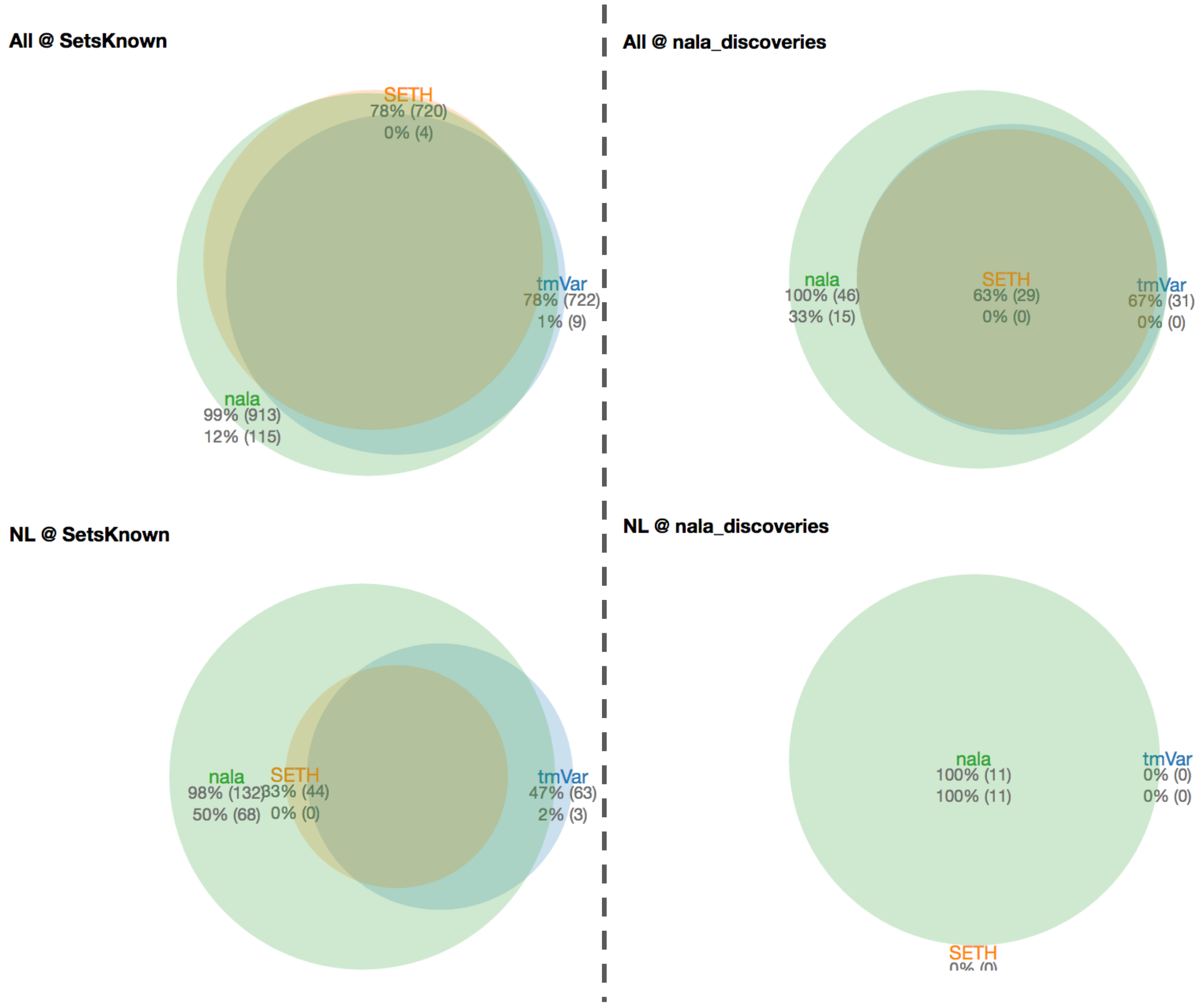

Fig. S1: *nala* could fully replace other methods, Venn Diagrams. Here, we looked at the following subset of all mentions. For each publication we considered all the mentions correctly identified by one of the top three methods and kept only the findings unique in each publication (the first sublabel after a method's name shows its correctly recovered percentage of unique mentions and, in parenthesis, the exact number of recovered unique mentions). We then asked the number of those had been identified uniquely by one of the methods distinguishing between all mentions and NL-only mentions (the second sublabel after a method's name shows its correctly recovered percentage of unique mentions that were not found by any other method and, in parenthesis, the exact number of recovered unique mentions that were not found by any other medhod). For instance, for all corpora containing publications of genes and proteins indexed in the databases (SetsKnown), 9 of the mentions (1%) were detected only by *tmVar* and 115 (12%) only by *nala*, while *SETH* found no mention in this data set that *nala* had not detected. One the other end, only *nala* correctly detected NL-only mentions in papers reporting discoveries on genes/proteins not indexed in databases, 11 (100%), right-bottwon Venn diagram.

Table S8. Detailed results of the effect on performance (*nala* method) of the word embedding (WE) features. * The possibly maximum StdErr for *nala_known* was taken from Supplementary Table S4 and the possibly maximum StdErr for *nala_discoveries* was taken from Supplementary Table S7.

	P	R	F	StdErr	Max StdErr*
nala_known					
ST WE=off	99	84	91	0	0
ST WE=on	99	88	93	0	0
NL WE=off	98	54	70	0	0
NL WE=on	93	75	83	0	0
nala_discoveries					
ST WE=off	93	52	67	1	4
ST WE=on	95	64	77	1	4
NL WE=off	100	3	5	0	9
NL WE=on	89	21	34	1	9

Table S9. Custom regular expressions we used to classify some one-letter-coded mutation mentions as standard form (ST). The regular expressions are here written in JavaScript –compatible form.

Regular Expression
\\w+: del [ACTGRNDEQHILKMFPSWYV] -?\\d+\\.\\.\\.-?\\d+
\\d+.*[ACTGRNDEQHILKMFPSWYV] --> [ACTGRNDEQHILKMFPSWYV]
[cgp]\\. ?\\d+ ?[ACTGRNDEQHILKMFPSWYV]>[ACTGRNDEQHILKMFPSWYV]

Table S10. List of software or utilities used with name, creation or version date, URL, and last accessed date.

Software Resources
2016. Online Mendelian Inheritance in Man, OMIM®. http://omim.org/. (2016/5/13 date last accessed)
NCBI. 2015. NCBI Text Mining Tools. http://www.ncbi.nlm.nih.gov/CBBresearch/Lu/Demo/tmTools/. (2016/5/13 date last accessed)
Okazaki, N. 2007. CRFsuite - A fast implementation of Conditional Random Fields (CRFs). http://www.chokkan.org/software/crfsuite/. (2016/5/13 date last accessed)
Stenetorp, P., Pyysalo, S. and Topić, G. 2014. Standoff format - brat rapid annotation tool. http://brat.nlplab.org/standoff.html. (2016/5/13 date last accessed)|
tpeng. 2015. tpeng/python-crfsuite. https://github.com/tpeng/python-crfsuite. (2016/5/13 date last accessed)

References

Jimeno Yepes, A. and Verspoor, K. Mutation extraction tools can be combined for robust recognition of genetic variants in the literature. *F1000Res.* 2014;3:18.
Thomas, P., *et al.* 2014. SETH - SNP Extraction Tool for Human Variations. https://rockt.github.io/SETH/. (2016/5/13 date last accessed)|.
Wei, C.-H., *et al.* tmVar: a text mining approach for extracting sequence variants in biomedical literature. *Bioinformatics* 2013;29(11):1433-1439.
